# Novel Therapies in Advanced Pancreatic Cancer: A Peak Beyond the Realms of Gene Targeting

**DOI:** 10.7759/cureus.89054

**Published:** 2025-07-30

**Authors:** Prashil Dave, Vishal Beriwala, Charmy Parikh, Anwar Uddin, Hiren Dayala, Raj H Patel, Punith Chowdary Chirumamilla, Andrew Winer

**Affiliations:** 1 Internal Medicine, State University of New York Downstate Health Sciences University, Brooklyn, USA; 2 Gastroenterology, Sardar Vallabhbhai Patel Institute of Medical Sciences and Research, Ahmedabad, IND; 3 Internal Medicine, Mercy Catholic Medical Center, Philadelphia, USA; 4 Surgery, University of Alabama, Birmingham, USA; 5 Internal Medicine, St. Mary Medical Center, Langhorne, USA; 6 Internal Medicine, Baptist Memorial Hospital, Oxford, USA; 7 Urology, State University of New York Downstate Health Sciences University, Brooklyn, USA

**Keywords:** advanced pancreatic cancer, bispecific antibody, gene targeting, pancreatic cancer treatments, tumor micro environment (tme)

## Abstract

Pancreatic cancer presents a formidable challenge in oncology, marked by its aggressive behavior and dismal prognosis. Despite a surge in global diagnoses, particularly in affluent nations, mortality data remain scarce in low-income countries due to limited access to diagnostic tools. The five-year survival rate remains dismally low, hovering around 6-8%, emphasizing the urgent need for innovative treatment strategies. In patients with resectable and borderline resectable pancreatic cancer, both neoadjuvant/perioperative mFOLFIRINOX and gemcitabine-nab-paclitaxel (GEM-Abraxane) have shown comparable median overall survival (~23-25 months) and R0 resection rates (~64-80%) in the SWOG S1505 trial. While mFOLFIRINOX demonstrates a modest progression-free survival benefit in metastatic settings, GEM-Abraxane remains a viable alternative, particularly in patients with borderline performance status. Metastasis, particularly in the lungs, is a common occurrence in pancreatic cancer, significantly impacting patient survival. Furthermore, bone metastasis, although less frequent, poses significant challenges and adversely affects outcomes. While the *KRAS* oncogene remains a dominant mutation, recent studies have identified additional actionable mutations (e.g., *BRCA1/2*, *PALB2*, and *NRG1* fusions) that offer potential targets for precision therapy. Bispecific antibodies, such as anti-CD3/mesothelin and CD3/claudin-18.2, have demonstrated potent cytotoxic activity in preclinical pancreatic cancer models and are progressing into early-phase clinical trials. Innovative therapies, such as combination regimens and precision medicine approaches, show promise in improving outcomes for patients with advanced pancreatic cancer. The efficacy of novel agents targeting specific molecular pathways, including CLDN18.2 and PDGFRα, is currently being evaluated in early-phase trials (e.g., CT041, IMAB362). Additionally, metabolic interventions and immunotherapies aimed at modulating the tumor microenvironment hold significant potential to enhance treatment efficacy. While pancreatic cancer remains a formidable adversary, recent advancements in therapeutic strategies offer hope for improving patient outcomes by leveraging cutting-edge technologies and fostering collaborative research endeavors. This literature review focuses on the latest clinical trial results of novel therapies that have emerged for the management of pancreatic cancer.

## Introduction and background

Pancreatic cancer, recognized for its aggressive nature and challenging prognosis, remains a significant concern in oncology [1]. In 2019, the American Cancer Society reported around 56,000 new pancreatic cancer cases in the USA, leading to an estimated 45,000 deaths, ranking it the third leading cause of death after lung and colorectal cancers [1]. Globally, pancreatic cancer stands as the seventh most fatal cancer for both genders, with approximately 459,000 new cases and 432,000 deaths based on GLOBOCAN 2018 estimates [2]. Forecasts suggest that pancreatic cancer might soon outrank breast cancer as the third leading cause of cancer-related deaths in the European Union [2,3]. The overall five-year survival rate for pancreatic cancer remains relatively low, typically around 6% to 8% [3].

Lung metastasis is common in pancreatic cancer, often occurring after liver metastasis, with an incidence reported as high as 45% [4]. Metastasis poses a substantial challenge in pancreatic cancer, with about half of the patients exhibiting distant metastasis upon diagnosis [5]. Pancreatic cancer often spreads to various areas, including bones, ranking as the third most common site for solid tumors to metastasize [6]. Bone metastasis can even be an initial sign of pancreatic cancer, impacting the prognosis and quality of life, especially in younger patients [6]. Bone involvement is associated with shorter median overall survival (OS) and increased risk of skeletal-related events, which significantly affect functional independence and pain control [6]. This spread to bones significantly affects survival rates, contributing to lower survival rates for pancreatic cancer patients with bone metastasis [6].

The *KRAS* oncogene, found in about 90% of pancreatic cancers, serves as a pivotal mutation driving the aggressiveness of the disease [7]. However, it is the combined impact of genes like *TP53*, *CDKN2A*, *SMAD4*, and newer findings in genes such as *KDM6A*, *RNF43*, *ARID1A*, and *BRAF*, among others, each with a frequency below 20%, that shapes its complex genetic landscape [8]. Mutations in *TP53* (50-75%), *CDKN2A* (30-40%), and *SMAD4* (30%) are among the most recurrent, contributing to disease progression, immune evasion, and resistance to therapy [8]. Comprehensive genetic profiling becomes crucial for understanding and addressing the various mutations that drive pancreatic cancer [8]. Distinct mutations, such as those in *GNAS*, and subsets with germline *BRCA* mutations highlight the intricate genetic makeup of this cancer [8]. These foundational mutations define the major oncogenic pathways of PDAC (pancreatic ductal adenocarcinoma) and inform the design of biomarker-guided therapeutic trials [8].

The shift towards targeting specific molecular mechanisms associated with pancreatic cancer has led to the emergence of novel therapeutic approaches [9]. Bispecific antibodies, capable of targeting multiple factors simultaneously, have emerged as promising treatments in this field [9]. CLDN18.2, identified as a distinctive protein, demonstrates expression across multiple cancer types such as gastric, gastroesophageal junction, breast, colon, liver, head and neck, bronchial, non-small-cell lung, and other gastrointestinal tract cancers [9]. Recent studies have shown that CLDN18.2 is aberrantly expressed in a subset of pancreatic ductal adenocarcinomas, making it an emerging target in ongoing early-phase therapeutic trials for pancreatic cancer [9]. However, treatment options for pancreatic cancer remain limited, with only modest improvements in survival over recent decades [9].

Neoadjuvant chemotherapy, particularly FOLFIRINOX, has demonstrated resection conversion rates of 25% to 40% in patients with borderline resectable pancreatic cancer [9]. However, even after curative-intent surgery, recurrence occurs in up to 80% of cases, highlighting the importance of both neoadjuvant and adjuvant systemic therapies in prolonging disease-free and OS [9]. The NALIRIFOX regimen, a combination of liposomal irinotecan with 5-FU, leucovorin, and oxaliplatin, has emerged as a promising first-line treatment for metastatic pancreatic cancer (mPC) [8]. Recent data from the NAPOLI-3 trial suggest improved progression-free and OS compared to gemcitabine (GEM) plus nab-paclitaxel (Nab-P), with a manageable toxicity profile [9]. However, its broader clinical applicability may be limited by increased gastrointestinal side effects and the need for careful patient selection due to performance status considerations [8]. There is an urgent unmet need for early diagnostic biomarkers, more effective systemic therapies, and strategies to overcome therapeutic resistance [9].

This review article explores the intricate molecular mechanisms underlying pancreatic cancer, providing insights into current standards of care for managing advanced or mPC. Furthermore, it navigates through recent trials and studies exploring diverse treatment modalities, including immune tumor microenvironment (TME) inhibition, metabolism-focused interventions, protein tropism (the selective affinity of specific proteins or therapies for tumor-specific markers), and the emergence of bispecific antibodies (engineered antibodies that bind two different antigens simultaneously to enhance tumor targeting and immune activation) in pancreatic cancer therapeutics. This discussion aims to unravel the evolving landscape of treatment strategies. It culminates in an exploration of potential breakthroughs poised to reshape the future of pancreatic cancer management, encompassing surgical interventions, radiation, chemotherapy, immunotherapy, and other innovative treatments on the horizon.

## Review

Methodology

We conducted an extensive search across PubMed (MeSH), EMBASE, and Google Scholar, spanning the last decade. We used the keywords 'advanced pancreatic cancer', 'management', or 'metastasis' and combined them using the Boolean operator 'and' with the words 'CPI-613', 'protein tropism', 'bispecific antibody', 'CAR-T', 'prophylaxis', and 'novel therapy'. We included studies comprising clinical trials, randomized controlled trials, and systematic reviews performed in humans only. Two reviewers independently reviewed articles. We excluded studies that did not meet these criteria. In total, we used 44 articles to write this review. The language included was English.

Genes involved in the progression of pancreatic cancer

Pancreatic cancer often originates from precancerous lesions known as pancreatic intraepithelial neoplasia (PanIN), where gene mutations accumulate over time, leading to dysplastic changes and eventual progression to cancer [10]. Most pancreatic cancers, approximately 90%, feature activating mutations in the *KRAS* oncogene, with specific mutations, such as G12D, G12V, and G12C, being the most prevalent [11]. These mutations hinder the normal function of *KRAS*, impacting various signaling pathways and contributing to the poor prognosis associated with pancreatic cancer [11]. However, the development of pancreatic cancer is not solely dependent on *KRAS* mutations; other genes, such as *TP53*, *CDKN2A*, and *SMAD4*, also play crucial roles in tumorigenesis and metastasis, with mutations detected in a significant proportion of pancreatic cancer cases [12].

Recent studies utilizing advanced sequencing methodologies have highlighted a series of genes harboring fresh mutations or modifications, each occurring at frequencies below 20% [13]. These genes encompass lysine demethylase 6A (KDM6A) at a rate of 18%, Rac family small GTPase 1 (RAC1) at 10%, ring-finger protein 43 (RNF43) at 10%, AT-rich interaction domain 1A (ARID1A) at 9%, and B-Raf proto-oncogene, serine/threonine kinase (BRAF) at 3% [13]. Additionally, mutations in TGF-β receptor 2 (TGFBR2) occur at 3%, mitogen-activated protein kinase kinase kinase 21 (MAP3K21) at 3%, switch/sucrose nonfermentable (SWI/SNF) at 3%, activin A receptor type 2A (ACVR2A) at 2%, activin A receptor type 1B (ACVR1B) at 2%, N-ras proto-oncogene, GTPase (NRAS) at 1%, family with sequence similarity 133 member A (FAM133A) at less than 1%, and zinc-finger matrin-type 2 (ZMAT2) at less than 1% [13]. Furthermore, large-scale sequencing studies have identified additional genes with mutations occurring at lower frequencies, as well as germline mutations, such as *BRCA*, associated with a small percentage of pancreatic cancer cases [14]. Mutations in genes like *GNAS*, particularly in intraductal papillary mucinous neoplasms (IPMNs), and various other genes related to cellular processes, including those involved in genome maintenance, contribute to the complexity of pancreatic cancer development [15]. Additionally, noncoding mutations in transcriptionally active regions of the genome are implicated in pancreatic cancer progression, highlighting the multifaceted genetic landscape underlying this disease [16].

Known standards of care for advanced/metastatic pancreatic cancer include GEM, a well-established antimetabolite and deoxycytidine analog, which has been a significant treatment in advanced pancreatic cancer (APC) for over 20 years [17]. Despite its long-standing use, early studies have shown only modest improvements in OS and quality of life compared to alternative treatments, such as bolus 5-fluorouracil (5-FU) [17]. Subsequent efforts to enhance outcomes through combination regimens often fell short, with many failing to demonstrate significant benefits, though some exhibited borderline efficacy at the expense of increased toxicity [18]. One notable combination, GEM + Nab-P, yielded significant improvements in both OS and progression-free survival (PFS) compared to GEM alone [18]. However, concerns persist regarding toxicity, particularly hematological adverse effects [19]. Other systemic options, such as FOLFIRINOX (FFX) monotherapy and GEM plus erlotinib or GEM plus cisplatin/fluoropyrimidine, as well as FOLFOX, show promise in specific patient subsets, albeit with varying levels of toxicity and effectiveness [20-23]. Ongoing research endeavors continue to explore additional combination therapies and sequencing approaches to optimize outcomes for pancreatic cancer patients [23]. The choice between systemic regimens, such as FOLFIRINOX and GEM-based therapies, is often guided by patient performance status, with FOLFIRINOX being preferred in patients with good functional status due to its increased efficacy, albeit with greater toxicity. In contrast, GEM remains an option for those with limited tolerance [22,23]. Furthermore, the identification of germline BRCA mutations informs the potential use of platinum-based therapies and maintenance with PARP inhibitors, reflecting a precision medicine approach as recommended by the National Comprehensive Cancer Network (NCCN) and the European Society for Medical Oncology (ESMO) guidelines [23,24]. Incorporating these factors into clinical decision-making enables the delivery of tailored therapy that optimizes benefits while managing adverse effects.

Trials and studies on drugs targeting the immune tumor microenvironment

Several clinical trials are underway to investigate novel treatments for metastatic pancreatic cancer. One such trial, conducted at MD Anderson Cancer Center, is studying the effectiveness of pembrolizumab in combination with the CXCR4 antagonist BL-8040 [24]. These agents enhance the body's immune response against cancer cells and inhibit tumor growth by blocking essential enzymes [24]. In this trial, combining BL-8040 with pembrolizumab in metastatic pancreatic cancer patients who had previously failed chemotherapy, treatment was generally well-tolerated, with some patients demonstrating partial response or stable illness [24]. Following BL-8040 treatment, peripheral blood samples showed higher quantities of circulating lymphocytes, decreased regulatory T cells, and enhanced expression of activation markers on T cells [24]. Furthermore, the combination therapy altered the tumor microenvironment by increasing T-cell infiltration and reducing the amount of immunosuppressive myeloid-derived suppressor cells (MDSCs) [24]. These data indicate that the BL-8040 and pembrolizumab combination therapy may potentially overcome the immunosuppressive environment of pancreatic cancer [24]. Similarly, a first-in-human study by Gossamer Bio Inc. assesses the potential of the CD11b modulator, GB1275, alone or combined with an anti-PD-1 antibody or standard of care in patients with metastatic pancreatic adenocarcinoma [25].

Another trial, led by the Parker Institute for Cancer Immunotherapy, evaluates the efficacy of different drug combinations, including APX005M with nivolumab, GEM, and Nab-P [26]. This study aims to determine the effectiveness of these combinations in treating metastatic pancreatic adenocarcinoma [26]. The study's primary goal was to assess the safety of combining APX005M, an agonistic CD40 monoclonal antibody, with GEM and Nab-P, with or without nivolumab, in patients with metastatic pancreatic adenocarcinoma [26]. The key objective was to determine the appropriate Phase II dose of APX005M for this patient population [26]. APX005M works by activating the CD40 receptor on immune cells, which causes T-cell-dependent tumor regression [26]. The paired treatment showed clinical effectiveness, with 58% of evaluated patients experiencing positive responses [26]. The treatment regimen was generally well-tolerated, but treatment-related side effects, such as a reduction in lymphocyte count and anemia, were frequent [26]. Notably, two chemotherapy-related deaths occurred due to adverse events [26]. Overall, the findings suggest that the combination therapy holds promise as a potential alternative to standard chemotherapy-only regimens for metastatic pancreatic adenocarcinoma, warranting further investigation in later-phase trials to validate its efficacy and safety [27]. Johns Hopkins is conducting a study involving epacadostat, pembrolizumab, and CRS-207, targeting patients who have progressed on prior chemotherapy [28]. The primary objectives include establishing the recommended dosage of epacadostat and assessing survival rates in both treatment groups [28].

Moreover, researchers at the University Health Network, Toronto, are investigating the potential of durvalumab and oleclumab in various advanced cancers, including pancreatic ductal adenocarcinoma, non-small-cell carcinoma, and squamous cell carcinoma of the head and neck [29]. This study aims to identify biomarkers predicting treatment response and understand the molecular effects of the drug combination [29]. Additionally, Arcus Biosciences, Inc. is conducting a Phase I trial evaluating AB680 in combination with zimberelimab, Nab-P, and GEM, while AB Science is comparing the efficacy and safety of masitinib in combination with GEM to placebo in patients with locally advanced or metastatic pancreatic cancer experiencing disease-related pain [30,31]. These trials represent a concerted effort to improve outcomes for patients with metastatic pancreatic cancer through innovative therapeutic approaches [31].

While the outcomes and mechanisms of trials involving BL-8040 + pembrolizumab, GB1275, APX005M, and durvalumab/oleclumab are promising, it is important to interpret these results within the context of each study. Direct cross-trial comparisons should be avoided, as variations in study design, patient selection, and endpoints can significantly impact efficacy and safety outcomes. Careful consideration of these factors is necessary to assess the therapeutic potential of tumor microenvironment-targeted agents accurately.

Trials and studies on drugs targeting metabolism

A Phase III study led by Cornerstone Pharmaceuticals aims to compare the efficacy and safety of FFX versus CPI-613 + modified FFX (mFFX) in patients aged 18 to 75 years with metastatic pancreatic adenocarcinoma [32]. Meanwhile, the Institut de Recherches Internationales Servier is conducting a Phase I study to establish the maximum tolerated dose (MTD) of AG-270, either alone or in combination with taxane-based chemotherapy, in individuals with advanced solid tumors or lymphoma exhibiting homozygous deletion of methylthioadenosine phosphorylase (MTAP) [33]. The trial evaluated the efficacy and safety of combining CPI-613 with mFFX with FFX in treatment-naïve patients with mPC [33]. CPI-613, a stable intermediate of a lipoate analog, targets pyruvate dehydrogenase and α-ketoglutarate dehydrogenase enzymes in cancer cell mitochondria, impacting critical metabolic processes [33]. The combination of CPI-613 with mFFX did not result in notable improvements in OS, PFS, or overall response rate (ORR) compared to FFX alone [33]. Both groups showed similar median durations for OS and PFS [33]. The safety profiles of the two therapy groups were identical, with significant side effects such as diarrhea, hypokalemia, anemia, neutropenia, thrombocytopenia, and fatigue [33]. However, the study did not find substantial therapeutic benefits of combining CPI-613 with mFFX over regular FFX treatment in mPC patients, despite CPI-613 targeting cancer cell metabolism [32].

Georgetown University is conducting a single-arm, multi-center Phase I trial to assess the safety and preliminary efficacy of eryaspase in combination with mFFX for patients with advanced pancreatic cancer [34]. Using a standard 3+3 design, the study aims to determine the MTD among four possible dose levels of eryaspase and evaluate safety parameters such as adverse events, vital signs, and laboratory tests [34]. The primary objective of the TRYbeCA-1 trial was to evaluate the efficacy and safety of eryaspase, a novel drug comprising asparaginase encapsulated within red blood cells, in conjunction with chemotherapy for patients with advanced pancreatic adenocarcinoma who had previously failed one systemic anti-cancer treatment [34]. Eryaspase works by breaking down asparagine and glutamine, which are vital for cancer cell growth and survival, thus perhaps improving the efficacy of chemotherapy [34]. Although the trial did not meet its main goal of enhancing OS when compared to chemotherapy alone, it displayed encouraging patterns, especially in a subset that received irinotecan/5FU treatment [34]. Eryaspase was well-tolerated, with tolerable side effects, including asthenia, diarrhea, and anemia, and did not exacerbate chemotherapy-related damage [35]. The results of this study indicate that eryaspase should be further researched, mainly when used alongside specific chemotherapy treatments, to assess its potential advantages in treating advanced pancreatic cancer [35]. Table 1 outlines clinical drug trials targeting the immune tumor microenvironment and metabolism.

**Table 1:** Clinical trials on drugs targeting the immune tumor microenvironment and metabolism G: gemcitabine; N: nab-paclitaxel; nivo: nivolumab; F: FOLFIRINOX; mF: modified FOLFIRINOX; MAS: masitinib; DCR: disease control rate; PFS: progression-free survival; ORR: objective response rate; OS: overall survival; HR: hazard ratio; PR: partial response; SD: stable disease; PD: progressive disease; DCR: disease control rate; AE: adverse events; SAE: Serious adverse events; MOS: months; DLT: dose-limiting toxicity; CI: confidence interval, G3: grade 3; NR: not reported, N/A: not available

Trial ID	Study Phase	Study Drugs	N	Outcomes Plus Results	Adverse Events	Status
NCT02907099 [24]	II	Pembrolizumab + CXCR4 antagonist BL-8040	20	SD: 2; PR: 1	N/A	Completed
NCT04060342 [25]	I&II	GB1275 with an Anti-PD-1 or nab-paclitaxel plus gemcitabine	61	SD: 6/19 (31.6%) in GB1275 monotherapy cohort; 9/16 (56.3%) in GB1275 + Pembroliumab cohort	Most frequent AE: photosensitivity reaction (20%), dysesthesia (13.3%), and pruritus (13.3%)	Terminated
NCT03214250 [26]	II	Gemcitabine, Nab-Paclitaxel, nivolumab, and APX005M (sotigalimab)	129	G+N+nivo: 1-year OS rate (95% CI): 0.577 (0.384 to 0.729); PFS (95% CI): 6.37 (5.19 to 8.80) G+N+APX005M: 1-year OS (95% CI): 0.481 (0.309 to 0.634); ORR (95% CI): 66.7 (22.28 to 95.67) G+N+nivo+APX005M: OS (95% CI): 0.413 (0.244 to 0.575); ORR (95% CI): 66.7 (22.28 to 95.67)	G+N+nivo: Elevated LFTs 24 (67%), thrombocytopenia 18 (50%) G+N+APX005M: CRS 9 (24%), elevated LFTs 30 (81%), thrombocytopenia 21 (57%) G+N+nivo+APX005M: CRS 12 (34%), elevated LFTs 26 (74%), thrombocytopenia 22 (63%)	Completed
NCT04104672 [30]	I/Ib	AB680, Zimberelimab, nab-paclitaxel, and gemcitabine	165	PR: 3 patients. CR of a target lesion: 1, SD: 5	Fatigue: (6, 43%), anemia (4, 29%), neutropenia (4, 29%)	Active, not recruiting
NCT03766295 [31]	III	Masitinib & gemcitabine	384	MAS-G: median OS of 3 mos (97.5% CI (11.0;18.0)). HR 0.46 (97.5% CI (0.2;0.9))	One AE or SAE: 96.3% and 9.1% respectively for MAS-GEM (n=246) vs 99.3% and 21.3% for palacebo-GEM (n=136)	Completed
NCT03504423 [32]	II	CPI-613, mFOLFIRINOX, FOLFIRINOX	528	CPI-613, mF: OS (95% CI): 11.10 mos (10.22 to 12.94)	CPI-613 plus mFFX vs. FFX arm: diarrhea (11.2% vs. 19.6%), hypokalemia (13.1% vs. 14.9%), anemia (13.9% vs. 13.6%), neutropenia (11.2% vs. 14.0%), thrombocytopenia (11.6% vs. 13.6%), and fatigue (10.8% vs. 11.5%)	Completed
NCT04292743 [34]	I	Eryaspase, FOLFIRINOX	19	PR: 24% (N=4); SD: 65% (N=11); PD: 11% (N=2) had progressive disease. DCR: 89%. PFS: 6.4 mos (95% CI 3.21–16.79). OS: 10.1 mos (95% CI 7.18 – NR)	G3 AEs: hypokalemia (22%), fatigue (11%), anemia (6%), hypotension (6%), diarrhea (6%), syncope (6%), and atrial fibrillation (6%)	Active, not recruiting

Trials and studies on protein tropism and bispecific antibodies

NCT03086369: Protein Tropism - Olaratumab

In this Phase Ib clinical trial, the safety and antitumor data of olaratumab, a monoclonal antibody specific for platelet-derived growth factor receptor alpha (PDGFRα), in combination with Nab-P + GEM, were studied for first-line metastatic pancreatic cancer patients [36]. A 3+3 dose escalation study was conducted with stage four pancreatic cancer patients divided into two cohorts: 15 mg/kg (cohort one) and 20 mg/kg (cohort two) [36]. Nab-P + GEM (125 mg/m²/1000 mg/m²) was administered in both cohorts with doses on days one, eight, and 15 of a 28-day cycle [36]. Major inclusion criteria were histologic or cytological diagnosis of adenocarcinoma of the exocrine pancreas that is metastatic (stage four) and not amenable to curative resection, absence of prior systemic treatment for metastatic disease, with allowance for adjuvant or neoadjuvant chemotherapy or radiotherapy completed ≥ 3 months ago with no lingering toxicities, presence of measurable lesions according to RECIST 1.1 criteria, and life expectancy of at least three months [36]. Major exclusion criteria were patients who received other first-line treatment, patients with prior treatment with Nab-P, and patients with current CNS or hematologic malignancy or metastasis [36]. The most reported adverse events were fatigue (50%), neutropenia (50%), nausea (46%), thrombocytopenia (41%), and constipation (32%) [36]. Both dose levels were tolerated, and the safety profile was consistent with Nab-P + GEM chemotherapy [36]. Most side effects were manageable by dose adjustments [36].

NCT03269526: Bispecific Antibody (BsAb)

BsAbs possess two unique binding domains, enabling simultaneous targeting of two antigens or epitopes on the same antigen [37]. BsAbs stand alongside monoclonal antibodies (mAbs) in therapeutics [37]. Targeting dual antigens or epitopes induces diverse physiological or antitumor responses akin to a combination of two mAbs [37]. Despite being a single molecule, they offer the benefits of cocktail therapy, potentially amplifying treatment outcomes synergistically [37].

This Phase Ib/II trial evaluates the safety, optimal dosage, and efficacy of anti-CD3 x anti-epidermal growth factor receptor (EGFR)-bispecific antibody-armed activated T-cells (EGFR-BATs) in treating participants with locally advanced or metastatic pancreatic cancer [37]. EGFR-BATs, comprising T cells coated with cetuximab and OKT3 bispecific antibodies, target tumor cells expressing EGFR, potentially leveraging the immune system to eradicate these cells [37]. Primary outcomes include the incidence of adverse events from the beginning of treatment until at least 30 days following the last study treatment and OS within a timeframe until subjects' death or study closure (whichever comes first), for an average of 36 months from study treatment completion [37]. The major inclusion criteria were histological or cytological confirmation of pancreatic adenocarcinoma with locally advanced or metastatic presentation, receipt of at least one dose of chemotherapy, regardless of treatment response, ECOG performance status of 0 or 1, LVEF of at least > 45%, and life expectancy of at least three months [37]. Major exclusion criteria were known hypersensitivity to cetuximab or other EGFR antibodies, a diagnosis of immunodeficiency or receipt of chronic systemic steroid therapy or other immunosuppressive therapy within seven days before the first study intervention, and recent treatment with any investigational agent within 14 days before the first study intervention [37]. In Phase I, dose escalation involved three weekly infusions of 1, 2, and 4 x 1010 BATs/infusion, followed by a booster infusion at three months for a total of 8 x 1010 BATs [37]. Phase II consisted of biweekly infusions of 1010 BATs/infusion over four weeks for 8 x 1010 EGFR-BATs [37]. Two patients had a complete response when chemotherapy was restarted after BATs [37]. Seventeen evaluable patients had a median OS of 31 months [37]. This trial concluded that the infusion of BATs is safe and induces endogenous adaptive antitumor responses [37]. It stabilizes disease, leading to improved OS, and there is evidence indicating it can induce antitumor activity and immunosensitize tumors to subsequent chemotherapy [37].

NCT03816163: Zolbetuximab + GEM and Nab-P

GEM and Nab-P treatment for mPC, which has a poor prognosis (<5% five-year survival), necessitates new therapies [38]. Claudin 18.2 (CLDN18.2), expressed in normal gastric cells and maintained in gastric cancers and some non-gastric carcinomas like pancreatic cancer, is targeted by zolbetuximab [38]. This chimeric IgG1 monoclonal antibody binds to CLDN18.2, inducing tumor cell death via immune mechanisms [38]. This Phase II study aimed to validate the recommended Phase II dose (RP2D) of zolbetuximab alongside Nab-P + GEM, evaluate OS, and gauge the safety and tolerability of this combined treatment, as well as tumor markers and pharmacokinetics (PK) of zolbetuximab, Nab-P, and GEM, and health-related quality of life [38]. Primary outcomes include the incidence of dose-limiting toxicities (up to 28 days); OS, defined as the time from randomization to death from any cause (up to 65 months); and safety, which was assessed by adverse events (AEs), defined as untoward medical occurrences related to product use (up to 65 months). Serious adverse events (SAEs) include outcomes such as death or hospitalization [38]. The study aims to evaluate the safety and effectiveness of GN alone or in combination with zolbetuximab in approximately 369 patients diagnosed with metastatic pancreatic cancer (mPC) exhibiting high levels of CLDN18.2 expression (moderate-to-strong staining in ≥75% of tumor cells) [38]. The trial incorporates a safety lead-in phase involving 3-12 patients to assess the tolerability of zolbetuximab (initial dose of 1,000 mg/m² on cycle one day one followed by 600 mg/m² every two weeks), utilizing a 3+3 design for dose escalation or de-escalation alongside GN [38]. Dose-limiting cytotoxicities will be assessed after cycle one and after confirming the recommended Phase II dose (RP2D) in the safety lead-in, approximately 357 patients will be randomized 2:1 into two arms: arm one receiving zolbetuximab Q2W on days one and 15 plus GN on days one, eight, and 15 of each cycle, and arm two receiving GN alone on days one, eight, and 15 of each cycle [38].

NCT03323944: CAR-T Against Mesothelin

This Phase I trial aims to assess the safety and feasibility of intravenous and local delivery of lentiviral-transduced huCART-meso cells in patients with unresectable/metastatic pancreatic adenocarcinoma [39]. Primary outcomes include the number of study subjects experiencing treatment-related adverse events, as defined by the NCI Common Terminology Criteria for Adverse Events. Secondary outcomes include PFS, OS, and objective response rate, all within a two-year timeframe [39]. The study is planned to be conducted in three cohorts [39]. The inclusion criteria for cohort one are histologically confirmed unresectable or metastatic pancreatic adenocarcinoma [39]. Cohort two comprises patients with histologically confirmed unresectable or metastatic pancreatic adenocarcinoma and either cytologically proven ascites or known peritoneal disease on radiologic imaging [39]. Cohorts three and four include patients with histologically confirmed unresectable or metastatic pancreatic adenocarcinoma with liver metastases as confirmed by pathology or radiographic imaging [39]. Patients with active invasive cancer other than pancreatic adenocarcinoma, HIV, hepatitis B, or hepatitis C infection, dependence on steroids, or patients using supplemental oxygen will be excluded from the study [39]. Cohort one (N=3-6) will receive a single intravenous infusion of 1-3x10^7^/m^2^ lentiviral-transduced huCART-meso cells on day 0 [39]. Enrollment will pause after the third subject has been enrolled for the formal dose-limiting toxicity (DLT) assessment [39]. If 0 DLT/3 subjects or 1 DLT/6 subjects, the study may advance to cohorts two and three [39]. For cohort four, up to six participants will receive a single dose of 1-3x10^7^/m^2^ huCART-meso cells intrahepatically following standard chemotherapy [39]. A one-week washout period will precede this administration [39]. Subsequently, participants may undergo up to two additional infusions of huCART-meso cells intravenously, maintaining the same dose level, with intervals of 21 to 42 days between infusions [39]. Cohorts two and three have been permanently closed due to feasibility concerns specific to the clinical/disease status of these patients at this stage of their treatment [39]. While these therapies highlight exciting directions in pancreatic cancer research, most remain in early-phase clinical trials or face feasibility limitations. As such, their current clinical applicability remains investigational, and larger, randomized studies will be critical to validate their therapeutic value [36-39]. These approaches represent an evolving frontier that may complement or eventually redefine conventional treatment strategies, but they are not yet practice-changing.

Among the trials discussed, three show especially promising early-phase signals: the bispecific EGFR-BATs (NCT03269526), the CD40 agonist APX005M combined with chemotherapy (NCT02706353), and the BL-8040 + pembrolizumab combination (NCT02826486). EGFR-BATs demonstrated a median OS of 31 months in a population with typically dismal survival outcomes [37]. APX005M demonstrated a 58% response rate in combination with GEM/Nab-P, indicating a meaningful synergy through CD40 activation [26]. The BL-8040 combination significantly modulated the tumor immune microenvironment, enhancing T-cell infiltration and reducing the number of MDSCs [24].

Table 2 displays clinical trials information regarding drugs with protein tropism and targeting specific genes.

**Table 2:** Clinical trials on drugs targeting protein tropism and specific genes N: nab-paclitaxel, G: gemcitabine, OS: overall survival, DLT: dose-limiting toxicity, BAT: bispecific antibody, CR: complete response, SD: stable disease, mos: months, mPFS: median progression-free survival, ALT: alanine aminotransferase, AST: aspartate aminotransferase, ALP: alkaline phosphatase, GGT: gamma-glutamyl-transferase, PC: pancreatic cancer, N/A: not available

Trial ID	Study Phase	Study Drugs	N	Outcomes Plus Results	Adverse Events	Status
NCT03086369 [36]	Ib	Olaratumab, nab-paclitaxel Gemcitabine	184	OS: Olaratumab + N + G (95% CI): 9.10 (7.49 to 14.09) mos. Placebo + N + G (95% CI): 10.81 (8.51 to 14.75) mos	DLT (grade 4 neutropenia): 1/12 (8.3%), fatigue (50); neutropenia (50%), nausea (46%), thrombocytopenia (41%), and constipation (32%)	Completed
NCT03269526 [37]	I&II	EGFR-BATs	20	SD: 4 patients for 6.1, 6.5, 5.3, and 36 mos. CR: 2 patients	N/A	Active, not recruiting
NCT03816163 [38]	II	Zolbetuximab + nab-paclitaxel + gemcitabine	369	N/A	N/A	Active, not recruiting
NCT03323944 [39]	I	huCART-meso cells	18	SD: 11/15; PFS: 2.1 mos	DLT: 1 (abdominal pain, jaundice). Low-grade fatigue and nausea were observed in 47% (7 out of 15) and 40% (6 out of 15) of patients	Recruiting
NCT04666740 [40]	II	Pembrolizumab, Olaparib	63	mPFS of 4 mos (2.1-5.4). mOS of 14 mos (10-NR)	Grade 3-5 AEs: total 5/14 (36%): 1 diarrhea (7%), 1 hyperglycemia (7%), 2 anemia (14%), 1 lipase increase (7%)	Recruiting
NCT03604445 [41]	I	BI 90567	37	SD: n = 13; (35%)	Grade ≥ 3 AEs: vomiting (11%), hyponatremia (8%), anemia (5%), diarrhea (5%), abdominal pain (5%), nausea (5%), hypokalemia (5%), pain (5%), and increased alkaline phosphatase (5%)	Terminated
NCT03600883 [42]	I	Sotorasib (AMG 510)	129	PR: 1 patient	Grade 3: elevated ALT (4.7%), elevated AST (2.3%), diarrhea (3.9%), anemia (3.1%), elevated ALP (1.6%), elevated GGT (0.8%), and hyponatremia (in 0.8%). Grade 4: elevated ALT 1 (0.8%)	Active, not recruiting
NCT02568267 [43]	I&II	Entrectinib (RXDX-101)	53	2 of 3 PC patients had a clinical response	Anemia 8 (12%), increased weight 7 (10%), increased blood creatinine levels 4 (6%), and fatigue 4 (6%)	Active, not recruiting
NCT02912949 [44]	II	Zenocutuzumab (MCLA-128)	40	N/A	N/A	Recruiting

## Conclusions

Pancreatic cancer poses a significant challenge in oncology due to its aggressive nature and poor prognosis, often spreading to the liver and lungs. Despite advancements in genetic profiling, identifying mutations such as *KRAS*, *TP53*, *CDKN2A*, and *SMAD4* that drive tumor formation, the overall five-year survival rate remains low. While GEM is the standard therapy, recent studies suggest that GEM + Nab-P may offer potential superiority, albeit with lingering concerns regarding toxicity. Promising avenues in treatment include the combination therapy of BL-8040 and pembrolizumab, which shows encouraging signs of overcoming the immunosuppressive microenvironment of pancreatic cancer. However, further investigations into optimal dosages and synergistic combination therapies are crucial to enhance efficacy while minimizing adverse effects. Monoclonal antibodies targeting the tumor microenvironment, when combined with chemotherapy, require careful exploration to optimize dosages and minimize toxicity. Additionally, the application of BsAbs has demonstrated the ability to enhance antitumor activity and sensitize tumors to subsequent chemotherapy, potentially improving OS rates. Targeted therapies tailored to individual tumor profiles, including personalized dosages and multi-drug regimens with reduced toxicity, hold significant promise for improving outcomes and extending the five-year survival rate for patients with advanced pancreatic cancer.

In conclusion, addressing the complex nature of pancreatic cancer requires a comprehensive approach integrating established and novel therapeutic strategies. Personalized treatment approaches tailored to the unique genetic characteristics of each tumor hold promise for enhancing patient outcomes and quality of life. It is important to recognize, however, that despite promising trial results, significant challenges remain in translating these advances into widespread clinical practice due to issues such as treatment accessibility, variability in patient response, and the need for further validation. By leveraging advanced technologies and fostering interdisciplinary collaboration, the landscape of pancreatic cancer care can be revolutionized, leading to better prognosis and patient care for this challenging disease.
